# 895 Time for New Definition of CLABSI: Trends of Bacteremia Patients in a Single Burn Center

**DOI:** 10.1093/jbcr/iraf019.426

**Published:** 2025-04-01

**Authors:** Jamie Hollowell, Lori Chrisco, Morgan Karlok, Booker King, Felicia Williams

**Affiliations:** University of North Carolina Jaycee Burn Center; University of North Carolina Jaycee Burn Center; University of North Carolina Jaycee Burn Center; University of North Carolina Jaycee Burn Center; University of North Carolina at Chapel Hill

## Abstract

**Introduction:**

Infections remain the primary source of complications and mortality in the burn population. Over the years there have been many updates to the infection prevention efforts as well as reimbursement for hospital acquired infections (HAIs). However, the burn patient remains a high-risk population. This population is often excluded from the data collection to form the definitions of HAIs like central-line associated blood stream infections (CLABSIs), yet these definitions do not consider the unique characteristics of this population. We sought to determine the characteristics of patients with bacteremia at our institution to help drive improvements in defining and preventing these life-threatening infections.

**Methods:**

Patients were identified using the Institutional Burn Center registry and linked to the clinical and administrative data. All adult patients admitted to the BICU with >10% TBSA burn between July 1, 2014, to June 30, 2024, were included. Demographics, length of stay (LOS), co-morbid conditions, and mortality were evaluated.

**Results:**

There was a total of 135 patients over the 10-year period with positive blood cultures. The patients were majority male 70.4%, average age was 50.8 years, average TBSA was 31.7%, 82.2% were flame burns. The average length of stay (LOS) was 93.5 days with an average ICU LOS of 56.1 days. The average for ventilator days was 51.7, average number of operative interventions was 4.8. The average days from admit to first positive blood culture was 26.0 days. Of these patients, 83.7% had a central line, 46.7% had renal failure, and 40.7% met the definition of multisystem organ failure (MSOF). Organism were stratified into gram-positive, gram-negative, and yeast/mold as causative agents. When stratified by organism, patients with yeast/mold had higher incidence of MSOF (p< 0.05) but mortality did not reach statistical significance.

**Conclusions:**

There continues to be a mismatch between the definition of and prevention measures available for the burn population. We note that many of our patients with bacteremia were male with flame injuries. Flame injuries and resultant contact to the environment during extinguishing maneuvers (stop-drop-roll, water hose, etc) can expose our patients to a variety of contaminants that we are unable to predict or plan for. Considering this exposure at time of admission and adjusting our prevention may improve outcomes. Additionally, adjusting definitions of these events may be necessary in terms of reimbursement and mortality indicators.

**Applicability of Research to Practice:**

This study reinforces the need for additional considerations of the criteria for HAIs in burn patients. This population has unique characteristics that fall outside the typical standards that may necessitate updates to definitions.

**Funding for the Study:**

N/A

